# The assessment of the impact of antiepileptic drugs on cognitive functions via N-200/P-300 potentials and neuropsychological measures

**DOI:** 10.1007/s10072-024-07606-5

**Published:** 2024-05-25

**Authors:** Javid Shafiyev, Ömer Karadaş

**Affiliations:** 1 Department of Neurology, Gülhane Training and Research Hospital, Etlik, Orgeneral Dr. Tevfik Sağlam Cd No:1, 06010 Keçiören/Ankara, Ankara, Turkey; 2 Department of Neurology, Gulhane Faculty of Medicine, Health Sciences University, Etlik, Orgeneral Dr. Tevfik Sağlam Cd No:1, 06010 Keçiören/Ankara, Ankara, Turkey

**Keywords:** Epilepsy, Antiseizure medications, Cognition, MoCA, Event-related potentials

## Abstract

**Objective:**

The effects of antiseizure medications (ASMs) on cognitive functions have not been fully elucidated. The primary aim of this study was to demonstrate potential changes in cognitive functions in patients diagnosed with epilepsy from both neuropsychological and electrophysiological perspectives. Our secondary objective was to assess the effects of administered ASM on cognitive functions by categorizing patients into different monotherapy and polytherapy groups.

**Materials and methods:**

A single-center, prospective patient registry study was conducted between May 2022 and June 2023. The inclusion criteria included epilepsy patients aged 18 to 50 years who were receiving ASM) treatment, either as inpatients or outpatients, and who did not have any syndromic diagnosis that may lead to cognitive disfunciton (such as primary progressive myoclonic epilepsies, Down syndrome and so on), and did not diagnosed previously or during examination that could affect dementia or cognitive functions. Patients who were scheduled to initiate new ASM treatment were evaluated using the Montreal Cognitive Assessment (MoCA) scale and Event-Related Potentials (ERP) assessment both before commencing treatment and three months thereafter.

**Results:**

A total of 320 participants were included in the study; 20 healthy controls and 300 epilepsy patients were included. Statistically significant differences were observed between the healthy control group and the epilepsy group in terms of average Montreal Cognitive Assessment (MoCA) scores and event-related potentials (ERPs) (n200, p300 latencies, n2p3 amplitudes) (*p<*0.05). Similarly, statistically significant differences were observed between the monotherapy and polytherapy groups in terms of average MoCA and ERP scores (*p<*0.05).

**Conclusion:**

This study demonstrated the detrimental effects of certain ASMs, particularly topiramate and carbamazepine, on cognitive functions. Furthermore, the negative impact on cognitive performance became more pronounced with an increasing number of concurrently used ASMs (polytherapy), with topiramate showing notable effects.

## Introduction

Epilepsy is a chronic neurological disorder characterized by recurrent epileptic seizures and associated somatic and psychiatric outcomes [3]. Indeed, epilepsy is one of the most prevalent and severe brain disorders affecting a substantial number of individuals worldwide. The highest risk for epilepsy development occurs in the early years of life, peaking in infants and young children. Furthermore, in older age groups, particularly in the elderly population, there is a second peak [21].

Cognitive impairments represent a common complaint among epilepsy patients [4], with an estimated 20-50% of patients experiencing cognitive issues [16]. The etiology of cognitive impairments in patients with epilepsy is multifactorial, with various contributors influencing their development and severity. The selection of ASMs for treatment can also impact cognitive functions. While many ASMs are effective at controlling seizures, some can lead to new neuropsychological disorders or worsening of existing cognitive conditions [4].

In particular, various methods have been developed to assess cognitive functions in individuals, especially for the identification of early signs of cognitive impairment and decline. The Montreal Cognitive Assessment (MoCA) is a commonly employed tool in clinical practice. Studies have indeed shown that the MoCA exhibits greater sensitivity in detecting cognitive decline than does the Mini-Mental State Examination (MMSE), another widely used clinical screening tool for Alzheimer's disease [9].

Electrophysiological markers of cognitive function Event-Related Potentials, namely, the N-200 and P-300 potentials, have been widely utilized and continue to be employed in scientific studies for the assessment and monitoring of cognitive functions. The N-200 potential emerges following a physical discrimination task or a semantic discrimination task. While physical discrimination occurs with passive attention, semantic discrimination necessitates selective attention. These potentials are also associated with pattern recognition and stimulus classification [7].

**Fig. 1:**
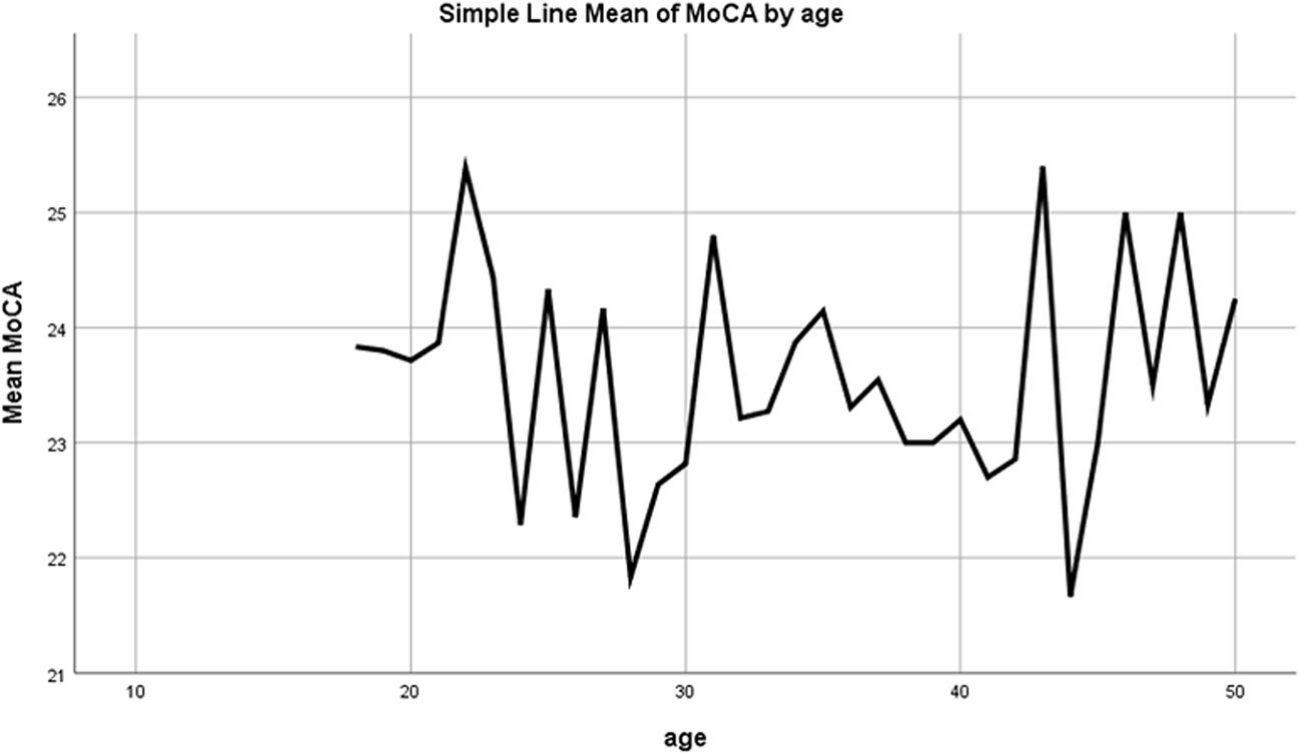
Linear regression analysis showing the effect of age on MoCA scores. MoCA: Montreal Cognitive Assessment. *The age has no effect on the MoCA score

The P300 manifests as a sizable, expansive (can be measure from both the anterior central (frontal) and posterior (parietal) regions of the scalp), positive component in the ERP, typically reaching its peak 300 ms or more after the onset of a rare, task-relevant stimulus. It exhibits a centro-parietal scalp distribution that is most pronounced above midline scalp sites [5]. Sutton et al. [24] first reported the P300 component, which is among the most extensively researched ERP components. It is commonly evoked within the oddball paradigm, where a haphazard sequence of stimuli is presented. These stimuli can be categorized into one of two groups, and the objective is to classify them, either by tallying or by pressing a button in response to stimuli belonging to a particular category. When stimuli from one of the categories are infrequent, often referred to as "oddballs," they trigger a P300 response (Fig. 1) [5, 14].

In recent years, significant progress has been made in the development and utilization of new generation ASMs. These novel medications have demonstrated considerable effectiveness in controlling epileptic seizures and are comparable to or even superior to traditional or older ASMs. Moreover, these patients often have a more favorable side effect profile, rendering them more tolerable and suitable for individuals with epilepsy. While numerous studies have investigated the effects of ASMs on cognitive functions, many of them rely on clinical data and neuropsychological tests, with limited inclusion of electrophysiological measurements in the literature.

Electrophysiological indicators closely linked to cognitive functions, such as N-200 and P-300 potentials, have not been extensively explored within the context of ASM treatment effects. Electrophysiological measurements, particularly the use of event-related potentials (ERPs), such as N-200 and P-300, can potentially detect subtle cognitive changes that may not be captured by conventional neuropsychological tests. These measurements have the potential to uncover nuanced cognitive alterations associated with ASM treatment effects.

Monitoring cognitive functions during the treatment of epilepsy patients and comprehending the influence of ASMs on cognitive functions are of paramount importance in terms of daily life activities. Research of this nature can provide valuable insights to enhance patients' quality of life and determine the most suitable treatment options.

The primary objective of this study was to demonstrate potential changes in cognitive functions in patients diagnosed with epilepsy from both neuropsychological and electrophysiological perspectives. Our secondary aim was to assess the effects of administered ASMs by categorizing patients into different monotherapy and polytherapy groups, thereby evaluating their impact on cognitive functions.

## Materials and methods

### Patients

Between May 2022 and June 2023, this study included patients aged 18 to 50 years who had been diagnosed with epilepsy, were using AMSs or were planning to start a new medication. The healthy control group consisted of individuals aged between 18 and 50 years without epilepsy or any diseases affecting cognitive functions (such as dementia, alcoholism, substance abuse, neurodevelopmental disorders, etc.) Written informed consent was obtained from all participants. The inclusion criteria for the study were age between 18 and 50 years, a diagnosis of epilepsy, current use of ASMs or planning to start a new medication. The exclusion criteria included being younger than 18 years or older than 50 years, receiving a diagnosis of psychogenic nonepileptic seizures, and having a diagnosis of cognitive disfunciton or dementia. The rationale behind setting the upper age limit at 50 for inclusion in the study stems from the increased risk of mild cognitive impairment (MCI), particularly beyond the age of 50, despite not enrolling patients with dementia [6]. By defining these inclusion and exclusion criteria, the study aimed to ensure that participants fell within a specific age range and possessed a confirmed diagnosis of epilepsy. The exclusion of individuals with specific conditions, such as psychogenic nonepileptic seizures, cognitive disfunction, or dementia, led to a more homogeneous group, enabling the research to focus on individuals with epilepsy who were either commencing or currently using ASMs. This approach contributes to the validity of the study and allows for a more targeted analysis of the cognitive effects of ASMs within this specific population.

### Tests

#### Montreal cognitive assessment (MoCA)

Developed in 2005 as a cognitive screening tool to detect mild cognitive impairment (MCI), the MoCA has since gained widespread use and is particularly recognized for its high sensitivity and specificity in the elderly adult population. The MoCA covers a wide range of cognitive functions, including visuospatial/executive function, naming, episodic memory, attention, language, abstraction, and orientation.

#### Event-related potentials (ERPs)

ERPs are small electrical voltages time-locked to specific events or stimuli that are elicited during EEG recordings; these ERPs represent the responses of brain structures to these events [20]. These time-dependent voltage changes, obtained in response to sensory, motor, or cognitive events, provide a noninvasive and safe method to investigate the psychophysiological underpinnings of mental processes. These findings offer insight into the neurophysiological correlates of various cognitive functions without requiring invasive procedures.

ERPs were recorded in a neurophysiology laboratory using a standard protocol and the Medelec EMG-EP device. Individuals were subjected to ERP recording in a quiet room with their eyes closed to minimize external distractors. The recording of ERPs was conducted at specific electrode points along the midline following the 10–20 system. Specifically, ERPs were recorded from the Fz, Cz, and Pz electrode points. A ground electrode was placed on the Fpz point, and reference electrodes were positioned on the mastoids to establish the recording setup. Additionally, a channel was placed on the infraorbital region (IO) to reduce potential artifacts from eye blinks.

Electrode impedance was maintained below 5 KΩ to ensure accurate signal recording. A filter range of 0.5 to 50 Hz was set for signal processing to help filter out unwanted frequency components. The analysis window for ERP recordings was set to 1000 ms to allow comprehensive evaluation of the brain's response to auditory stimuli. The sound intensity was set above the established threshold value by 40 dB. While the sound intensity was generally set at 80 dB, individual variations were observed, and adjustments were made accordingly for each participant.

By adhering to this standardized recording protocol, the study aimed to obtain reliable and consistent ERP data, allowing for a comprehensive assessment of the brain's response to auditory stimuli.

To ensure participants' full comprehension of the counting process, a one-minute counting task was administered before the test began. The study employed an auditory oddball paradigm to record auditory event-related potentials (ERPs). During the ERP recording, participants were presented with two types of auditory stimuli through headphones: nontarget stimuli (frequent, 3000 Hz) and target stimuli (rare, 2000 Hz). The average auditory threshold for the stimuli was set at 80 dB. The stimuli were presented to both ears at a frequency of 0.7 Hz, meaning that auditory tones were delivered 0.7 times per second.

While participants were instructed to count the target tones (2000 Hz), nontarget tones (3000 Hz) were also presented. The target tones (2000 Hz) were randomly presented at a rate of 20%, while nontarget tones (3000 Hz) were presented randomly at an 80% rate. Throughout the recording session, a total of 40 averages of target tones were collected without any artifacts. To ensure data reliability and consistency, the test was repeated twice.

For the analysis of potentials, the amplitude was calculated from the N200 peak to the P300 peak, often referred to as the N200/P300 amplitude. Additionally, the latencies of N200 and P300 potentials were calculated considering the midpoint of each potential. The statistical analysis of the study was conducted using recordings obtained from the Cz electrode (CZ-A1 channel), a specific electrode point along the midline according to the 10–20th edition.

## Methods

This single-center prospective study included patients receiving inpatient and outpatient treatment between May 2022 and June 2023. The data were collected through face-to-face interactions in a clinical setting and an electrophysiology laboratory.

Patients who were using ASMs and those planning to initiate a new medication in monotherapy were administered the Montreal Cognitive Assessment (MoCA) test, a neuropsychological test, by a neurologist. On the same day as the MoCA test, cognitive functions were evaluated neurophysiologically using N-200 and P-300 signals in the hospital's neurophysiology laboratory. Patients newly prescribed monotherapy were requested to return for a follow-up visit three months later. During this follow-up visit, they were required to complete both the MoCA test and their evoked potentials (N-200, P-300) were recorded.

These procedures were conducted within a clinical setting and an electrophysiology laboratory, aiming to evaluate cognitive functions comprehensively through both neuropsychological and neurophysiological assessments. The study was designed to track potential changes in cognitive functions over time, especially in patients newly treated with ASMs.

## Statistical analysis

In the present study, statistical analysis was performed using the IBM SPSS Statistics 26 software package, a commonly used tool for data analysis in various research fields. A significance level (alpha) of 0.05 was used to determine statistical significance. For categorical data evaluation, such as sex or treatment group, frequencies and percentages were calculated to illustrate the distribution of the data. Descriptive statistics, including the mean, standard deviation, minimum, and maximum values, were calculated for the numerical data to summarize central tendency and variability.

To compare dependent variable data, such as pre and post-treatment MoCA scores, P300 and N200 latencies, and N2P3 amplitudes, a paired-samples t test was utilized. Before the T test, it was observed that the data conformed to a normal distribution by examining skewness and kurtosis values, which indicated that the data were normally distributed. This test is appropriate for analyzing repeated measurement data taken from the same individuals, such as measurements before and after treatment. One-way ANOVA and post hoc analysis were used to compare MoCA and ERP data among the different groups (healthy control group, epilepsy group (all participants except for the healthy controls)), and treatment groups: monotherapy (total number of individuals using monotherapy), polytherapy (total number of individuals using polytherapy). Before conducting the post hoc analysis, a test of homogeneity of variance was performed, and it was observed that the variances were homogenous among the groups. Consequently, Tukey analysis was conducted

The selected statistical methods were chosen to appropriately assess and compare various aspects of the data, enabling the study to draw meaningful conclusions about the effects of different treatments on cognitive functions**.**

Additionally, linear regression analysis was conducted to investigate the relationship between age and the measurements. Linear regression is employed to model the relationship between a dependent variable (in this case, measurement values) and one or more independent variables (such as age) to determine if a linear relationship exists between them.

Ethical approval was obtained from the Clinical Research Ethics Committee. All participants provided informed and voluntary consent by signing the informed consent form.

## Results

### Sociodemographic characteristics

The study included a cohort comprising 300 patients and 20 healthy subjects (constituting the control group). The mean age of the participants was 35.4 (±13) years, with the healthy group exhibiting an average age of 34.6 (±13.9) years and the patient cohort averaging 35.44 (±16.3) years. Among the participants, 50.6% (162) were male, while 49.4% (158) were female. Notably, the sex distribution was 45% (9) male and 55% (11) female within the healthy volunteer cohort, whereas the patient cohort displayed a distribution of 51% (154) male and 49% (146) female. Comprehensive details regarding the participants' age and sex distributions can be found in Table 1 for reference**.**

**Table 1:** Demographic data

Variables	Total (*n* = 320)	Healthy Group (*n* = 20)	Patient Group (*n* = 300)	Monotherapy Group (*n* = 100)	Polytherapy Group (*n* = 200)
Age, years, Mean ± SD	35.4 (13)	34.6 (13.9)	35.4 (16.3)	35.3 (12.3)	35.4 (13.3)
Gender, male n (%)	162 (50.6)	45 (9)	51 (154)	58 (58)	95 (47)

Mean ± SD: mean ± standard deviation

The distribution of ASMs utilized by the patients is presented in Table 2. In accordance with the treatment protocols, the patients were categorized into two main groups: monotherapy and polytherapy. Furthermore, patients were subdivided into subgroups based on the specific ASMs and medication combinations they were administered (Tables 3, 4,5 and 6).

**Table 2:** Distribution of ASMs

Subgroups (*n*=300)	N *n* (%)	Gender Male *n* (%)	Age (Mean±SD)
Levetiracetam	22 (7.3)	13 (59)	34.8 (13.4)
Carbamazepine	20 (6.6)	11 (55)	35.3 (12.7)
Lacosamide	19 (6.3)	13 (68.4)	35,9 (13.2)
Lamotrigine	20 (6.6)	11 (55)	35 (11.6)
Topiramate	19 (6.3)	10 (52.6)	35.7 (11.6)
Levetiracetam + Lacosamide	20 (6.6)	10 (50)	36.2 (14.2)
Levetiracetam + Carbamazepine	21 (7)	10 (47.6)	36.4 (13.1)
Levetiracetam + Topiramate	19 (6.3)	8 (42.1)	35 (12.9)
Levetiracetam + Lamotrigine	20 (6.6)	8 (40)	34.5 (12.1)
Levetiracetam + Carbamazepine + Topiramate	20 (6.6)	10 (50)	36.5 (13.6)
Levetiracetam + Carbamazepine + Lacosamide	22 (7.3)	11 (50)	34.2 (12.3)
Levetiracetam + Carbamazepine + Lamotrigine	20 (6.6)	12 (63.2)	36.8 (15.4)
Levetiracetam + Lacosamide + Lamotrigine	19 (6.3)	12 (63)	34,2 (11,7)
Levetiracetam + Lamotrigine + Topiramate	19 (6.3)	8 (42.1)	36.1 (14.7)
Levetiracetam + Lacosamide + Topiramate	20 (6.6)	8 (40)	34.5 (14.6)

Mean ± SD: Mean ± standard deviation

**Table 3:** Relationships between main groups with event-related potentials and MoCA scores

Main Groups	P300		N200		N2P3		MoCA Score	
	Mean±SD (ms)	P	Mean±SD (ms)	*P*	Mean±SD (uV)	*P*	Mean ± SD	*P*
Healthy	320.5 (9)		220.3 (9.2)		12 (1.2)		26 (1.2)	
Epilepsy	337 (12)	<0.05*	236.8 (11.8)	<0.05*	9.8 (1.1)	<0.05*	22.9 (1.1)	<0.05*
Healthy	320.5(9)		220.3 (9.2)		12 (1,2)		26 (1.2)	
Pretreatment epilepsy group	328.3 (6)	<0.05*	228.8 (6.3)	<0.05*	10.7 (0.6)	<0.05*	24.7(1.2	<0.05*
Healthy	320.5 (9)		220.3 (9.2)		12 (1.2)		26 (1,2)	
Monotherapy	334 (11)	<0.05*	233.9 (10.4)	<0.05*	10.3 (0.9)	<0.05*	23.8 (1.1)	<0.05*
Healthy	320.5 (9)		220.3 (9.2)		12 (1.2)		26 (1,2)	
Polytherapy	342.8 (12)	<0.05*	241.4 (12.1)	<0.05*	9.4 (1.1)	<0.05*	22.7(1)	<0.05*
Monotherapy	334 (11)		233.9 (10.4)		10.3 (0,9)		23.8 (1.1)	
Polytherapy	342.8 (12)	<0.05*	241.4 (12.1)	<0.05*	9.4 (1.1)	<0.05*	22.7 (1)	<0.05*
Monotherapy	334 (11)		233.9 (10.9)		10.3 (0.9)		23.8 (1.1)	
Dual Medication	334.8(12)	1.000	234.7 (12.1)	1.000	10 (1)	0.963	23.2 (1.2)	0.403
Monotherapy	334 (11)	<0.05*	233.9 (10.9)		10.4 (1)		23.8 (1.1)	
Triple Medication	349.3 (10)		250 (12)	<0.05*	8.8 (1)	<0.05*	22.2 (1)	<0.05*

Mean ± SD: mean ± standard deviation, ms: millisecond, μV: microvolt, ; MoCA: Montreal Cognitive Assessment

* The difference between the healthy group and the epilepsy group was statistically significant

* The difference between the healthy group and the epilepsy group not receiving medication was statistically significant

* The difference between the healthy group and the monotherapy and polytherapy groups was statistically significant

* The difference between the monotherapy group and the polytherapy group was statistically significant

* The difference between the monotherapy group and the triple medication group was statistically significant

**Table 4:** Effect of Medication Subgroups in the Monotherapy Group on MoCA Scores and ERPs at 3 Months of Treatment

Monotherapy Subgroups	(ERPs) Pre-treatment and 3rd-month values						Pre-treatment and 3rd Month MoCA Score	
	P-300		N-200		N2P3			
	Mean± SD (ms)	*P*	Mean± SD (ms)	*P*	Mean± SD (uV)	*P*	Mean± SD	*P*
Levetiracetam	328.8;327	0.856	228.7; 228.1	0.863	10.7;10.6	0.709	24.5(1); 24.4(1.1)	0.593
Lamotrigine	328.3; 329	0.893	228.4; 227	0.796	10.9;10.8	0.964	24.3 (0.9); 24.6(1)	0.721
Topiramate	327.2;347.1	<0.05	226;245	<0.05	10.7:9.1	<0.05*	24.4(1);21.7 (1.4)	<0.05*
Lacosamide	329.8;330.6	0.703	229.2;230.5	0.584	10.7;10.8	0.553	24.4(0.9);24.3 (1.4)	0.865
Carbamazepine	327.2;337.4	<0.05	226;235.8	<0.05	10.6; 9.4	<0.05*	24.5(1);23.1(1.2)	<0.05*

Mean ± SD: mean ± standard deviation; MoCA: Montreal Cognitive Assessment; , ERP: event-related potentials, ms: millisecond, μV: microvolt

* The difference between the MoCA scores before medication and at the 3rd month for the Topiramate group is statistically significant

* The difference between the MoCA scores before medication and at the 3rd month for the Carbamazepine group is statistically significant.

* The difference between the P300 and N200 latencies and N2P3 amplitudes before and after 3 months of medication treatment in the Topiramate group was statistically significant

* The difference between the P300 and N200 latencies and N2P3 amplitudes before and at 3 months of medication treatment in the Carbamazepine group is statistically significant

**Table 5:** Distribution of MoCA scores in triple and dual medication subgroups

Triple and Dual Medication Subgroups	MoCA Score	
	Mean±SD	*P*
LEV+CBZ+LCM LEV+CBZ+LTG	22.5 (1,1) 23.1 (1)	0.446
LEV+CBZ+LCM LEV+LCM+LTG	22.5 (1.1) 24 (0.9)	<0.05
LEV+CBZ+LCM LEV+CBZ+TPM	22.5 (1.1) 19 (0,9)	<0.05
LEV+CBZ+LCM LEV+LTG+TPM	22.5 (1.1) 21.1 (1.2)	<0.05
LEV+CBZ+TPM LEV+CBZ+LTG	19 (0.9) 23.1 (1)	<0.05*
LEV+CBZ+TPM LEV+LCM+LTG	19 (0.9) 24 (0.9)	<0.05*
LEV+CBZ+TPM LEV+LCM+TPM	19 (0.9) 21 (1.1)	<0.05*
LEV+CBZ LEV+LCM	23 (1.1) 24.7 (1.2)	0.04*
LEV+CBZ LEV+LTG	23 (1.1) 24.8 (1.1)	0.04*
LEV+CBZ LEV+TPM	23 (1,1) 21 (0,9)	<0.05*
LEV+LCM LEV+LTG	24.7 (1.2) 24.8 (1.1)	1.000
LEV+LCM LEV+TPM	24.7 (1.2) 21 (0.9)	<0.05*
LEV+LTG LEV+TPM	24.8 (1.1) 21 (0.9)	<0.05*

Mean ± SD: mean ± standard deviation; MoCA: Montreal Cognitive Assessment

* The difference between the Lev+Tpm group and the other dual medication groups is statistically significant

* The difference between the Lev+Cbz group and the Lev+Lcm and Lev+Ltg groups is statistically significant

* The difference between the Lev+Cbz+Tpm group and the other triple medication groups was statistically significant

**Table 6:** Relationships between triple and dual medication subgroups with event-related potentials (ERPs)

Triple and Dual Medication Subgroups	P300		N200		N2P3	
	Mean±SD (ms)	P	Mean±SD (ms)	P	Mean±SD (uV)	P
LEV+CBZ+LCM LEV+CBZ+LTG	341.7 (9) 335.3 (11.5)	0.09	242.4 (10) 235 (11.7)	0,008	9.0 (0.8) 9.8 (1.2)	0.09
LEV+CBZ+LCM LEV+LCM+LTG	341.7 (9) 333.3 (6.5)	0.04	242.4 (10) 232 (6.2)	0.03	9.0 (0.8) 10 (0.7)	0.04*
LEV+CBZ+LCM	341.7 (9) 355.5 (5)	<0.05	242.4 (10) 253.9 (7)	<0.05	9.0 (0.8) 8.1 (0.4)	<0.05
LEV+CBZ+TPM						
LEV+CBZ+LCM LEV+LTG+TPM	341.7 (11) 342 (9,3)	0.793	242.4 (11.3) 242.1 (9)	0.991	9.0 (1.3) 9.1 (1.1)	0.884
LEV+CBZ+TPM LEV+CBZ+LTG	355.5 (5) 335.3 (11,5)	<0.05	253.9 (7) 235 (11.7)	<0.05	8.1 (0.4) 9.8 (1.2)	<0.05*
LEV+CBZ+TPM LEV+LCM+LTG	355.5 (5) 333.3 (6,5)	<0.05	253.9 (7) 232 (6.2)	<0.05	8.1 (0.4) 10 (0.7)	<0.05*
LEV+CBZ+TPM LEV+LCM+TPM	355.5 (5) 346.4 (9)	0.04	253.9 (7) 245 (8.8)	0.03	8.1 (0.4) 9.1 (0.5)	0.04*
LEV+CBZ LEV+LCM	337.4 (9,1) 329.1 (11)	0.04	237.5 (10) 229.6 (10.9)	0.04	9.5 (0.8) 10.4 (1)	0.03*
LEV+CBZ LEV+LTG	337.4 (9.1) 328.4 (10)	0.02	237.5 (10) 228.2 (10.3)	0.03	9.5 (0.8) 10.8 (0.9)	0.02*
LEV+CBZ LEV+TPM	337.4 (9.1) 346.4 (8.7)	0.04	237.5 (10) 246.1 (8.2)	0.03	9.5 (0.8) 8.9 (0.5)	0.04*
LEV+LCM LEV+LTG	329.1 (11) 328.4 (10)	0.632	229.6 (10.9) 228.2 (10.3)	0.634	10.4 (1) 10.8 (0.9)	0.59
LEV+LCM LEV+TPM	329.1 (11) 346.4 (8.7)	<0.05	229.6 (10.9) 246.1 (8.2)	<0.05	10.4 (1) 8.9 (0.5)	<0.05*
LEV+LTG LEV+TPM	328.4 (10) 346.4 (8.7)	<0.05	228.2 (10.3) 246.1 (8.2)	<0.05	10.8 (0.9) 8.9 (0.5)	<0.05*

Mean ± SD: mean ± standard deviation, ERP: event-related potentials, ms: millisecond, μV: microvolt

* The difference between the Lev+Cbz+Tpm group and the other triple medication groups was statistically significant

* The difference between the Lev+Cbz+Lcm group and the Lev+Ltg+Lcm group was statistically significant

* The difference between the Lev+Tpm group and the other dual medication groups was statistically significant

* The difference between the Lev+Cbz group and the Lev+Ltg and Lev+Lcm groups is statistically significant

### MoCA test scores and the correlation with ASM usage

In terms of MoCA scores, the monotherapy group (23.8) is found to have higher scores compared to the polytherapy group (22.7) (*p<*0.05). Within polytherapy, both dual (23.2) and triple therapies (22.2) are determined to have lower MoCA scores compared to healthy controls (26) (*p<*0.05). The Moca scores were significantly lower in the epilepsy group (24.7) before starting treatment (montherapy) compared to the healthy group (26) (p < 0.05). The monotherapy group (23.8) is identified to have higher MoCA scores compared to polytherapy (22.7) (*p<*0.05).

#### Detailed comparisons within polytherapy

The Moca scores of the monotherapy group (23.8) were significantly higher than those of the polytherapy group using triple therapy (22.2), but there was no significant difference between the group using dual therapy (23.2) (*p*>0.05).

#### MoCA score changes with specific medications

Patients starting LEV, LTG, or LCM monotherapy show no significant changes in MoCA scores after three months (*p*>0.05). However, the TPM and CBZ groups showed a significant decrease in MoCA scores three months after treatment (*p<*0.05).

#### Detailed analysis of polytherapy

Subgroups with TPM (Lev + TPM) and CBZ (Lev + CBZ) have significantly lower MoCA scores than other subgroups (*p<*0.05). The MoCA scores were significantly lower in the group using Lev+TPM compared to the group using Lev+CBZ (*p<*0.05). There are no significant differences in MoCA scores between Lev + LCM and Lev + LTG subgroups (*p*>0.05).

#### Specific polytherapy combinations

Subgroups with both TPM and CBZ (Lev + TPM + CBZ) show lower MoCA scores than those without (*p<*0.05). Subgroups containing TPM (Lev + LCM + TPM and Lev + LTG + TPM) also show lower MoCA scores than those without (*p<*0.05).

### Event-related potentials (ERPs) in relation to the utilization of ASMs

In this study, it was observed that the P300 and N200 latencies of the healthy (320.5;220.5) control group were significantly shorter compared to both polytherapy (342.8; 241.4) and monotherapy (334; 233.9) groups (*p<*0.05). Similarly, the N2P3 amplitudes of the healthy control group (12) were significantly higher than those of both the polytherapy (9.4) and monotherapy (10.3) groups. Furthermore, statistically significant longer P300 and N200 lantencies and lower N2P3 amplitudes were detected among the polytherapy group (342.8; 241.4; 9.4) using dual (334.8; 234.7; 10) or triple therapies (349.3; 250; 8.8) compared with the healthy control group (320.5; 220.3; 12) (*p<*0.05).

#### Comparison of latencies and amplitudes among medication groups

When comparing the average latencies of P-300 and N-200 among the main groups of medication use, it was found that both the average latencies of P-300 and N-200 in the monotherapy (334; 233.9) group were significantly shorter than those in the polytherapy (342.8; 241.4) group (*p<*0.05). Similarly, the N2P3 amplitudes of the monotherapy (10.3) group were significantly higher than those of the polytherapy group (9.4) (*p<*0.05).

#### Detailed analysis of polytherapy subgroups

When we divided the polytherapy group into subgroups of patients using dual and triple medications, it was found that the N200 and P300 latencies of the monotherapy group were shorter than those of the triple therapy group. Similarly, the N2P3 amplitudes were significantly higher (*p<*0.05). However, no statistically significant difference was observed between the monotherapy group and the dual therapy subgroup (*p*>0.05).

#### Changes in ERPs with medication over time

Patients starting LEV, LTG, or LCM monotherapy show no significant changes in the average latencies of P-300 or N-200, or N2P3 amplitude, between before starting medication and after 3 months (*p*>0.05). However, significant prolongation in N200/P300 latencies and notable decreases in N2P3 amplitudes were observed three months after initiating TPM (327.2:347.1, 226; 245, 10.7; 9.1) and CBZ (327.2; 337.4, 226; 235.8, 10.6; 9.4) groups (*p<*0.05).

#### Impact of specific medications on ERPs

Furthermore, within the polytherapy group and considering subgroups based on the use of two medications, we found that the subgroups containing TPM (Lev + TPM) (346.4;246.1;8.9) and CBZ (Lev + CBZ) (337.4;237.5;9.5) exhibited statistically significant longer latencies of P-300 and N-200 compared to the other two-medication subgroup, as well as significant lower amplitudes in N2P3 amplitudes (*p<*0.05).

### Regression analysis of MoCA test scores

According to our regression analysis, neither the age nor the sex of the participants had a statistically significant impact on the MoCA test score; P-300 and N-200 latencies; or N2P3 amplitudes in both the healthy control group and all the other medication group subgroups (*p<*0.05).

## Discussion

In our prospective randomized study, the average MoCA score for epilepsy patients was 22.9. The MoCA scores of the epilepsy group were significantly lower than those of the healthy control group (p < 0.05). When we divided the epilepsy group based on medication usage, our study revealed that the average MoCA score of the polytherapy group was significantly lower than that of the monotherapy group (*p<*0.05). Within the polytherapy subgroup analysis, the subgroup receiving triple medication therapy had an even lower MoCA score (*p<*0.05).

No statistically significant differences were found in the average MoCA scores before and 3 months after medication initiation for patients using LEV, LTG, or LCM as monotherapy (*p*>0.05). However, patients taking CBZ and TPM as monotherapies exhibited statistically significant decreases in MoCA scores at the 3-month mark, which was particularly pronounced for topiramate (*p<*0.05).

There were significant differences in N200 and P300 latencies and N2P3 amplitude between epilepsy patients and healthy controls (*p<*0.05).

In our study, compared with patients in the monotherapy group, patients in the polytherapy group were found to have significantly longer P-300 and N-200 latencies and lower N2P3 amplitudes (*p<*0.05). When subdividing the polytherapy group into subgroups based on the use of two or three medications, it was observed that the three-medication subgroup exhibited longer N-200 and P-300 latencies and ower N2P3 amplitude.

When examining the ERP values of the subgroups within the monotherapy group before starting medication and 3 months after starting medication, patients using LEV, LTG, or LCM as monotherapy showed no statistically significant difference in their average values after 3 months (p > 0.05). However, for patients receiving TMP and CBZ monotherapy, a statistically significant difference was found in their ERP values, particularly for topiramate, after 3 months (*p<*0.05).

Current research, as evident in our study, suggests that the incidence of epilepsy is slightly greater in males than in females [2]. The average age of the epilepsy patients in our study was 35.4 years. Patients aged less than 50 years were included criteria, thereby reducing the likelihood of confounding factors related to mild cognitive impairment (MCI) and other types of dementia.

In a study conducted by Novak et al., the average MoCA score for the healthy control group was 27.5, while for the epilepsy group, it was 23.3 [12]. Like in our study, this research also revealed a statistically significant difference in the average MoCA score between epilepsy patients and healthy controls [12]. However, in the Novak et al. [6] study, most patients were in the monotherapy group, and the number of participants in the polytherapy group was smaller. This circumstance could reasonably contribute to the higher average MoCA scores in our study.

Witt et al. reported that LEV did not have a significant impact on cognitive functions [23]. Additionally, Kossoff et al. mentioned that although LEV might lead to side effects such as depression, anxiety, and aggression in patients, these side effects tend to occur at lower rates than other ASMs [10].

In the study conducted by Ogunjimi et al., which evaluated the effects of LEV and CBZ monotherapies on cognition in women with epilepsy, significant differences were found in cognitive function between the CBZ and LEV groups across various domains. Specifically, patients using CBZ had weaker overall language, memory, and attention scores, whereas the LEV group exhibited higher average scores in comprehension and fluency [13]. These three studies, including ours, have identified similar findings concerning cognitive functions.

In our study, among the monotherapy groups, the TPM group exhibited the most significant decrease in MoCA scores after treatment. It is well known in the literature that TPM is an ASM with a recognized detrimental effect on cognitive functions. Jette et al. reported that patients using TPM had a two- to fivefold greater risk of developing cognitive complaints than patients using a placebo. However, interpreting these results requires consideration of differences in study design and patient populations. Jette et al.'s study included TPM as an adjunctive treatment with rapid titration for refractory patients, which might have contributed to a greater risk of cognitive complaints [8]. In contrast, Arroyo et al. reported that cognitive complaints decreased to approximately 3-4% when TPM was administered as monotherapy. This inconsistency in findings could be attributed to differences in patient selection and treatment approach between the two studies [1].

Smith et al. reported on a study involving 81 patients with refractory focal seizures and reported that LTG was not associated with any adverse cognitive effects [18]. In fact, numerous studies comparing LTG with other ASMs have clearly indicated that LTG is less associated with cognitive impairments [11]. Similarly, our study did not observe any negative impact of LTG on cognition.

Moreover, none of the studies in the literature, like ours, have evaluated medication-specific medication regimens, such as monotherapy, polytherapy, dual medication treatment, or triple medication treatment. However, certain data, such as years of education, duration of epilepsy, and frequency of seizures, were not assessed in the methodologies of the mentioned studies. This limitation is inherent to our study.

Sarić Jurić et al. reported a statistically significant difference in the average P-300 latencies and N2P3 amplitudes between their control group (composed of healthy participants) and their epilepsy group (measured at 313 ms, 340 ms, 13.5 μv, and 9.5 μv, respectively) [17]. While the results of our study and the research by Sarić Jurić et al. are similar in terms of significance, the slight differences in P-300 latencies and N2P3 amplitudes in participants in our study could be attributed to cultural and/or educational factors. Like in our study, Sarić Jurić et al. reported that the average P-300 latencies and N2P3 amplitudes in the polytherapy group were significantly different (in the negative direction) from those in the monotherapy group (measured at 348.5 ms, 323 ms, 12.1 μV, and 9.3 μV, respectively) [17].

Soysal et al. reported similar N-200 and P-300 latencies in healthy control and epilepsy groups in our study [19]. Öztürk et al. also investigated the effects of TPM on cognitive functions and reported similar results [15].

Although there is no specific publication in the literature that evaluates ERPs before and 3 months after LEV treatment, a study by Tumay et al. reported a P-300 latency of 328 ms in patients using LEV, which is similar to our study [22]. Our findings are in line with the literature suggesting that LEV does not have a significant impact on cognitive functions [23].

When we examined the average P-300 and N-200 latencies and N2P3 amplitudes of the CBZ monotherapy group before treatment and 3 months after treatment, we found a statistically significant difference between the average values before and after treatment (*p<*0.05). In a study by Tumay Y et al. that evaluated the effects of LEV, CBZ, and VPA on cognition, the average P-300 latency for patients using CBZ was 383 ms. Although the average P-300 latency for CBZ users (383 ms) was longer than that in our study (335 ms), the association between CBZ and poorer cognitive function compared to LEV supports the findings of our study in this context [22].

There are limited studies in the literature that have investigated the effects of LCM on cognition; in particular, studies evaluating ERPs are lacking. Our findings, which did not show a significant difference in ERP latencies or amplitudes before and after LCM treatment, support the literature suggesting that LCM does not have a negative impact on cognitive function.

Regarding the effects of TPM on cognition, the most recent study by Saric Juric J et al. reported that patients using TPM had an average P-300 latency of 352 ms and an N2P3 amplitude of 10.8 μV [17]. These results are statistically similar to our study's findings.

A study by Saric Juric et al. [17] reported average P-300 latency values of 341.5 ms and N2P3 amplitude values of 9.2 μV for patients receiving LTG monotherapy. These values appear to be higher than the values we observed in our study. However, similar to our findings, this study reported that LTG monotherapy resulted in the shortest P-300 latency. The results of our study are in line with numerous pieces of literature suggesting that LTG does not have a negative impact on cognition [11].

When stratifying patients into monotherapy and polytherapy cohorts, it was discerned that individuals within the polytherapy cohort manifested inferior cognitive performance relative to their counterparts in the monotherapy cohort. Within the confines of the monotherapy faction, notable cognitive repercussions were observed, primarily within the ambit of TPM and, to a conspicuous degree, with TPM and CBZ administration. Moreover, concomitant with Patients treated with polytherapy regimens inclusive of TPM and CBZ exhibited exacerbated cognitive debilitation. A salient cornerstone of our investigation resides in the discernment that, upon scrutiny of the MoCA and ERP datasets, LTG, LEV, and LCM exhibit enhanced cognitive efficacy compared with alternative antiseizure agents, spanning both monotherapeutic and polytherapeutic paradigms.

## Conclusion

In conclusion, comprehending the effects of medications or medication combinations utilized in treatment of cognitive dysfunction will facilitate the management of conditions such as epilepsy, which can detrimentally impact cognitive functions. Furthermore, this understanding can contribute positively to socioeconomic aspects by helping patients maintain their independence in daily life activities. Awareness of these effects can assist in providing enhanced care and support, enabling individuals to lead a satisfactory life despite their conditions.

A limitation of our study is that patients receiving polytherapy may be at risk for drug-resistant epilepsy, and this condition could negatively impact cognition over prolonged periods of multiple drug use. Considering this, we believe that not only the quantity of medication but also the duration of medication use should be included.
